# Tailored patient therapeutic educational interventions: A patient‐centred communication model

**DOI:** 10.1111/hex.13377

**Published:** 2021-11-24

**Authors:** Laetitia Ricci, Julie Villegente, Déborah Loyal, Carole Ayav, Joëlle Kivits, Anne‐Christine Rat

**Affiliations:** ^1^ CHRU‐Nancy, INSERM Université de Lorraine, CIC 1433 Clinical Epidemiology Nancy France; ^2^ Université de Reims Champagne‐Ardenne Reims France; ^3^ Université de Lorraine, APEMAC Nancy France; ^4^ University of Caen Normandie Caen France; ^5^ Rheumatology Department University Hospital Center Caen Caen France

**Keywords:** health communication, healthcare providers, interviews, six‐function model, tailored intervention, thematic analysis, therapeutic patient education

## Abstract

**Background:**

Tailoring therapeutic education consists of adapting the intervention to patients' needs with the expectation that this individualization will improve the results of the intervention. Communication is the basis for any individualization process. To our knowledge, there is no guide or structured advice to help healthcare providers (HCPs) tailor patient education interventions.

**Objectives:**

We used a data‐driven qualitative analysis to (1) investigate the reasons why HCPs tailor their educational interventions and (2) identify how this tailoring is effectively conducted. The perspective aimed to better understand how to individualize therapeutic patient education and to disentangle the different elements to set up studies to investigate the mechanisms and effects of individualization.

**Design:**

Individual semistructured interviews with 28 HCPs involved in patient education were conducted. The present study complied with the COREQ criteria.

**Results:**

Why individualization is necessary: participants outlined that the person must be thought of as unique and that therapeutic education should be adapted to the patient's personality and cognitive abilities. The first step in the individualization process was formalized by an initial patient assessment. Several informal practices were identified: if needed, giving an individual time or involving a specific professional; eliciting individual objectives; reinforcing the relationship by avoiding asymmetrical posture; focusing on patients' concerns; leading sessions in pairs; and making the patient the actor of decisions.

**Conclusion:**

From our thematic data analysis, a model for tailoring patient education interventions based on the Haes and Bensing medical communication framework is proposed. The present work paves the way for evaluation, then generation of recommendations and finally implementation of training for individualization in educational interventions.

**Short Informative:**

Tailoring in therapeutic education consists of an adaptation to patients' needs. Communication is the basis for any individualization process. There is no model of patient‐centred communication in educational interventions. From semistructured interviews with HCPs, we propose a patient‐centred communication model for tailoring patient education intervention.

## 1 INTRODUCTION AND BACKGROUND

In 1998, the World Health Organization described therapeutic patient education (TPE) as ‘educational activities essential to the management of pathological conditions, managed by healthcare providers (HCPs), duly trained in the education of patients and designed to help a patient (or a group of patients and their families) to manage their treatment and prevent avoidable complications, while keeping or improving their quality of life’.^1^ TPE covers organized activities, including psychosocial support, designed to make patients fully aware of their disease and to inform them about care, hospital organization and procedures as well as health‐ and disease‐related behaviours.^2^ TPE can be a way to cope with change in self‐identity and to plan, pace and prioritize.^3^ Educational activities can include interventions based on health education, promotion of medical adherence, illness‐related problems in everyday life, promotion of physical activities, psychological support and social counselling (see e.g., Meng et al.^4^). Several meta‐analyses showed that TPE interventions are beneficial for patients, but they are rarely described in detail.^5^, ^6^, ^7^, ^8^, ^9^, ^10^ In 2007, Conn even spoke about the ‘black box’, whereby no one can determine what actually happened during an intervention,^11^ which is not appropriate from a strictly epistemological point of view.

A description of the intervention is essential to understand its mechanisms and to explain the obtained results. TPE interventions are complex by nature because they are based on multiple components such as the variability of individuals, HCPs, healthcare systems, economics, political factors and their interaction.^12^, ^13^, ^14^

Adult patient education has its own particularities. Indeed, the people to whom it is addressed already have representations and experiences and have built up knowledge. TPE interventions often question their certainties. Although education includes a part of transmission of knowledge or know‐how, it also aims at the appropriation of this knowledge and its transformation by the person to whom it is transmitted. TPE interventions place a strong emphasis on experiential learning. The patient's reflexivity is emphasized to define their own goals and action plans. It also aims to develop self‐knowledge and critical thinking skills that contribute to the ability to make choices and to exist in a social environment.^15^

The individualization process consists of adapting the intervention to a patient's needs, according to assessment on admission and subsequent re‐evaluations.^16^ To achieve this process in the context of TPE, several levers are already envisioned, for example, taking into account factors such as cultural context^17^, ^18^, ^19^ or patients' health literacy level.^20^, ^21^ The importance of social workers or psychologists is also highlighted.^22^, ^23^ For individualization, professionals should have precise and up‐to‐date medical knowledge of the illness and high communication skills reinforced by training courses.^24^, ^25^, ^26^ Patient initial assessment is the first step of TPE from which individualized education can be operationalized and deployed.^22^, ^26^, ^27^, ^28^, ^29^ More generally, for Hawkins et al.^30^ ‘Tailoring refers to any of a number of methods for creating communications individualized for their receivers, with the expectation that this individualization will lead to larger intended effects of these communications’. For Hawkins et al., health communications can be segmented into three levels: mass communication (standardized content), targeted communication (constitution of homogeneous groups to deliver an adapted content) and tailored communication (individually tailored intervention with an adapted content to individual needs). According to Petty and Cacioppo's Elaboration Likelihood Model of Persuasion,^31^ people will more carefully consider the elements of a message and thoughtfully process information if they perceive it to be personally relevant.

The principles of using tailored communication states that by tailoring content, superfluous information is eliminated. People pay more attention to information that they perceive to be personally relevant, and this information process is more likely to have an effect. Information that addresses the unique needs of a person will be useful in helping them become and stay motivated and will promote desired life‐style changes.^32^ Comprehension is expected to improve, and exchanges on the content and changes in behaviours and attitudes will be enhanced.

Hawkins et al.^30^ highlight the need to focus research on how tailoring works (i.e., which strategies are relevant for achieving tailoring goals).

Communication between patients and HCPs is the basis for any individualization process in TPE interventions. Thus, tailoring requires effective efficient communication behaviour. Research on medical communication^33^, ^34^, ^35^ helps in understanding the complexity of clinical situations in which physicians understand the individual characteristics and the emotional, cognitive and behavioural responses of the patient to respond appropriately.

Although patient–physician communication assessment instruments are available in the literature,^36^, ^37^, ^38^ nonmedical HCP communication is little developed. It seems unlikely that models developed in the field of medical communication can be applied to nurses' and allied health professionals' communication with patients without any adaptation. For example, in 2016, the Communication Skills Training module on how to respond empathically to patients was developed for inpatient oncology nurses.^39^ In 2018, the COMFORT communication curriculum was developed for nurses and has become the first theoretically grounded and evidence‐based curriculum for teaching palliative care communication.^40^ Finally, there are few developments on patient‐centred communication for women with breast cancer.^41^ In addition, training in communication skills for nurses is emerging,^39^, ^42^, ^43^, ^44^, ^45^, ^46^, ^47^ which highlights needs in this domain.

We used a data‐driven qualitative analysis to (1) investigate the reasons why HCPs tailor their educational interventions and (2) identify how this tailoring is effectively conducted. The perspective was to better understand how to individualize TPE interventions and to disentangle the different elements of individualization to set up studies to investigate the mechanisms and effects of individualization.

This study was part of the Classification of Patient Therapeutic Education Programs Components (CONCErTO) project, whose objective is to identify the institutional, organizational, pedagogical, psychosocial and medical element components of a patient education programme affecting the outcome, participation and sustainability of the programme through qualitative investigation (ClinicalTrials.gov Identifier: NCT02717182). The objective of the CONCErTO project is to provide a tool to describe TPE interventions in research publications and participate in understanding their mechanisms of action. Better insight may help HCPs build effective and generalizable interventions in the clinical context.

## 2 METHODS

### 2.1 Study design

The present study was carried out in compliance with the COREQ criteria.^48^ Semistructured individual interviews were conducted with HCPs involved in TPE.

### 2.2 Study setting, sample and recruitment

#### 2.2.1 Programme selection

Programmes were chosen among Lorraine Regional Health Agencies‐authorized programmes from their updated list. After eliminating 23 programmes for children, spleen diseases and psychiatric disorders, 113 programmes remained. We selected 12 programmes in the Lorraine region and two outside the region: one in Paris (Île‐de‐France Region) and one in Grenoble (Auvergne Rhone Alpes Region).

Sample heterogeneity is considered essential to capture in depth a large and diverse content. To construct a maximum variation sampling,^49^, ^50^ we identified three key dimensions of variations of the programmes:^51^, ^52^ diseases; hospital or nonhospital programmes; and urban or rural programmes.

We chose the following programme types:

1. Programmes for diseases: digestive cancers (*n* = 2), cardiovascular diseases (*n* = 2), kidney failure (*n* = 2), rheumatic diseases (*n* = 1), chronic obstructive pulmonary disease (*n* = 1), asthma (*n* = 1), chronic pain (*n* = 1), multiple sclerosis (*n* = 1), hepatitis (*n* = 1), diabetes/obesity (*n* = 1) and multiple pathologies (*n* = 1).
2. Hospital programmes (*n* = 9), nonhospital programmes (*n* = 5).
3. Urban programmes (*n* = 10) and rural programmes (*n* = 4).

The list of the Regional Health Agencies included the name and contact information of the programme manager (public data). L. R. and J. K. phoned the programme manager to explain details and ask them to participate in the CONCErTO project. No manager refused programme participation.

### 2.3 Participants

Interviews were conducted where programmes took place by an experienced female health PhD psychologist (L. R.) and a female experienced PhD sociologist (J. K.) from April 2016 to May 2017. No one else was present besides the participant and the researcher during the interview.

We interviewed all HCPs involved in each programme who were present on the day of the interview. The researchers did not have any relationship with HCPs before the start of the study. All solicited HCPs agreed to participate after a short presentation of the CONCErTO project. The interview duration was about 1 h. Interviews were integrally audio‐recorded and transcribed.

### 2.4 Protocol development and data collection

During three meetings in the first quarter of 2016, an interview guide was designed with a clinician/epidemiologist (also coordinator of a TPE programme), a psychologist and a sociologist (also coordinator of a transversal TPE unit of a teaching hospital). The key research questions for discussion were to describe the TPE practices and identify elements that may affect the outcome, participation and sustainability of the intervention.

Probing questions for in‐depth exploration were organized based on May's normalization process theory,^53^ with three levels: macro (institutional elements), meso (elements linked to the TPE organization) and micro (elements linked to the patient and the HCP–patient relationship). Probing questions were defined to identify HCPs' perception of TPE (see Box 1) and to investigate the institutional (macro), organizational (meso) and pedagogical, medical and psychosocial (micro) components of a TPE programme affecting outcomes such as participation and sustainability of the programme. The key aim was to encourage HCPs to speak freely about factors of success or difficulty in TPE.

Box 1. **The interview guide for questions on therapeutic patient education (TPE)**

**Opening questions**

‘How do you practice TPE? What do you think makes TPE successful, what works? what is important? What makes TPE less successful?’

**Probing questions for in‐depth exploration**

1. Role of the institution

2. Organization/structure of the TPE

3. Integration in the department/network, support

4. Role of training

5. Methods, facilitation, HCP–patient relationship, pedagogical tools used, customization

6. Integration of theoretical aspects in patient education

7. Impact of patient characteristics

8. Impact of social factors

9. Other aspects not addressed during the time of interview

Abbreviation: HCP, healthcare providers.

Individualization was addressed with the probing question 5 on ‘methods, facilitation, HCP–patient relationship, pedagogical tools used, and customization’.

We collected demographic information of the participants (i.e., specific profession, age class and number of years of experience in TPE). For each included programme, we also collected the mode of delivery of sessions (one‐to‐one, in groups, both) and whether a specific strategy for composition of the participant groups was applied.

### 2.5 Analysis and rigour

We followed a general inductive approach^54^ to identify themes from the HCPs' discourse to properly capture their perception. Verbal elaborations from the probing question 5 were used to investigate the process of tailoring. After a careful and open reading of transcriptions, themes for thematic analysis were identified. First, a health psychologist (L. R.) read the three first interviews and proposed a first draft of a coding grid. Next, on the basis of two other interviews, the grid was refined and tested during a meeting with a sociologist (J. K.) and a clinician/epidemiologist (A. C. R.). At this step of exploring HCPs' views on TPE, tailoring appeared as a major element in TPE. Therefore, we chose to focus specifically on exploring individualization. A document specifying the content of the themes (elements to be included/excluded in each theme) was created and revised progressively to stabilize the coding grid. All themes and subthemes were defined and discussed during the meeting to triangulate the perspectives of psychology, sociology and clinical and public health. In this type of triangulation, researchers provide different insights for a deeper and broader understanding of findings.^55^, ^56^

L. R. and J. K. are experts in qualitative research, A. C. R. is also a coordinator of a TPE programme and J. K. is also a coordinator of a transversal TPE unit of a teaching hospital. The variety of expertise ensured rich data interpretation.^57^ In 2018, J. V. was trained in the coding grid. Six interviews were double‐coded by L. R. and J. V. (21% of the qualitative material). All coding disagreements between the two coders were resolved by discussion. In case of persistent disagreement, resolution was obtained by discussion with a third researcher (A. C. R.). Then, we measured the level of agreement between the two coders using Cohen's *κ* coefficient. This calculation provided an overview about the process of achieving coder consensus.^58^ After reaching a *κ* value of 0.89, J. V. encoded the remaining data. Throughout the coding process, difficulties and queries were regularly discussed by the whole team.

The recruitment process ended after data saturation, that is, on obtaining sufficient data to report on all aspects of the phenomenon.^59^, ^60^ Data saturation is achieved when concepts and subconcepts cannot be further specified with additional data.

To achieve data saturation, L. R. conducted interviews with HCPs until the information redundancy point was reached (no emergence of new idea from data). J. K. then conducted three more interviews to ensure data saturation (one with an allied health professional and two with a nurse). In other words, we continued data collection for three more interviews to confirm that no new relevant themes emerged from supplementary interviews.^61^ Data analysis was performed using NVivo v11.

A committee of experts was organized with two health psychologists (L. R. and D. L.), a sociologist (J. K.) and a clinician/epidemiologist (A. C. R.) to identify from themes that emerged in qualitative data analysis (1) the reasons why HCPs wanted to individualize TPE and (2) how HCPs effectively tailored their interventions.

### 2.6 Ethical considerations

The protocol was approved by the Comité de Protection des Personnes (CPP (Sud Est 1)) (no. ID‐RCB: 2017‐A00247‐46, CPP no.: 2017‐12). All participants provided their written and oral consent.

## 3 RESULTS

Saturation was achieved with 28 interviews (20 nurses, 6 dieticians, 1 physiotherapist and 1 psychologist). Table 1 shows that sampling was varied to collect a large diversity of perceptions concerning TPE. The average education experience was 7 years.

**Table 1:** Description of HCPs according to disease addressed by the therapeutic patient education

Disease	No. of programmes	HCP professions	Age group (years)	Years of education experience
Digestive cancers	2	Nurse	21–30	6
		Dietician	21–30	0.3
Cardiovascular diseases	2	Nurse	51–60	18
		Nurse	51–60	12
		Dietician	31–40	3
Kidney failure	2	Nurse	61–70	8
		Nurse	61–70	8
		Nurse	41–50	8
		Nurse	51–60	8
		Dietician	51–60	10
		Dietician	51–60	6
Rheumatic diseases	1	Nurse	51–60	12
COPD	1	Nurse	31–40	6
Asthma	1	Nurse	31–40	2.5
		Nurse	51–60	6
Chronic pain	1	Nurse	61–70	3
		Nurse	21–30	3
		Nurse	21–30	1
		Nurse	21–30	2
Multiple sclerosis	1	Nurse	51–60	10
Hepatitis	1	Nurse	31–40	6
Diabetes/obesity	1	Nurse	51–60	6
		Nurse	41–50	13
		Dietician	21–30	6
		Psychologist	51–60	10
Multiple pathologies	1	Nurse	41–50	8
		Dietician	41–50	8
		Physiotherapist	41–50	8

Abbreviations: COPD, chronic obstructive pulmonary disease; HCPs, healthcare providers.

Table 2 shows that among the 14 programmes, most (*n* = 10) proposed group sessions. One programme proposed group sessions and an individual session and three programmes proposed only individual sessions.

**Table 2:** Types of patients' proposed sessions in TPE programmes

Groups	Individuals	Groups plus individuals	Total
10	3	1	14

Abbreviation: TPE, therapeutic patient education.

The objectives of two of the three programmes with only individual sessions were to improve safety skills because training did not involve any kind of tailoring in skills achievement (parenteral nutrition in digestive cancers and injections in multiple sclerosis). These addressed skills can, indeed, not be compromised without endangering the patient. Nevertheless, adjustments could be made in the acquisition process.

If the patient wants to go slower, we will go slower. (Participant, 1)

In one other programme for hepatitis, individual sessions were proposed for confidentiality reasons because the target population was often former drug users.

One single programme included both individual and group sessions.

In one programme, significant others were systematically invited to participate in the last group session. In three other programmes, significant others' participation for group sessions was left to the discretion of the patients. The HCPs' perceived interest was ‘to have a better understanding of each other’ (Participant, 12).

Table 3 presents an overview of why and how an educational programme is tailored in an individual education configuration or in a group intervention without any strategy concerning group composition.

**Table 3:** Synthesis of qualitative results of therapeutic patient education (TPE) programmes

Themes/subthemes	Illustrative quotes
**Why?**	
**To take care of a patient not just a disease**	‘We take the person as a whole, with his/her environment, disease (s), desires, needs, tastes, and then we adapt the TPE’ (p. 23).
**To adapt to personal functioning**	‘With the patient profile it's difficult and it's up to me to adapt, but this very rigorous patient, it's absolutely not possible for me to tell him you can move forward or backward 24 hours because he won't be well’ (p. 20).
**To adapt to cognitive and language abilities**	‘We use a vocabulary that is not the same for everyone and approach things in different ways’ (p. 16).
**How? Take time**	‘To get there, the key is to take time’ (p. 20).
**Organization of the programme**	
Conduct an initial assessment	‘Initial assessment allows us to find the most appropriate program to their needs. If you have a person who is physically hyperactive, it's not the most urgent to make him meet the group workshops with the physiotherapist’ (p. 22).
Systematically add an individual session	‘We tell them: you measure your breath for a month, you send us the results and then we meet again for a last session, for crisis management’ (p. 14).
Add alternative or complementary individual sessions if needed	‘Because he has questions that are too personal and it doesn't concern the theme of the day's workshops… we let the group go and then we resume in a somewhat informal way for some questions’ (p. 9).
Involve an HCP not systematically solicited if needed	
**Content of the programme**	
Define objectives along the sessions' progression	‘Patients always leave [the session] with an objective’ (p. 12).
Consider the patient's lifestyle	‘If the person gets up at 9 am, they will not be asked to measure their blood sugar at 7 am’ (p. 26).
Adapt content and activities	‘This involves adapting the content to age and age‐related concerns’ (p. 12).
Take into account pedagogical assessment	‘Not to continue the sessions if there are things that have not been understood, to be able to readjust before continuing the session’ (p. 14).
Individualized follow‐up between sessions	‘For the most vulnerable people, I also do telephone follow‐up’ (p. 4).
**Relationship with the patient**	
Allow for a deeper relationship than in routine practice	‘I'm not here as a care prescriber (…) saying you have to, you have to, you have to’ (p. 28).
Built over time	It is important that patients ‘always deal with the same professional’ (p. 5).
**Communication style with patients**	
Avoid asymmetric positions	‘We are no more with the image of the nurse who is there for her knowledge, who bombard patients with things to do’ (p. 9).
Concentrate on patients' interests	‘We have to focus communication on the patients' interests and not unpack everything we know’ (p. 4).
**How to facilitate group sessions**	
Know the participants beforehand	‘As I know them well, when I animate I know very well who I have to look at, who I have to tell, why I am going to ask such a patient to give an example or how he feels’ (p. 4).
Facilitate sessions in pairs	‘In pairs, better listening to the group where sometimes someone will take the floor, the floor will be cut. We have trouble hearing the two people, and it is true that the second person can therefore reformulate what has been said next door to take up etc. Uh, all alone, I think there might be more forgotten remarks (…) that you don't necessarily hear alone when you are in a conversation’ (p. 2).
**Patient actor of the individualization process**	The patient can choose to address ‘questions or problems that have not been perceived’ (p. 17).

### 3.1 Why individualization is needed?

#### 3.1.1 TPE takes care of a patient, not just a disease

HCPs emphasized the holistic approach of TPE, which takes into account the whole person in their environment at a given time. All study participants outlined that, in TPE, the person must be thought of as unique.

#### 3.1.2 Need to adjust to the patient's personality

To optimize TPE outcomes, HCPs adapt their communication to the singular functioning of the patient. Different personal functioning required an adapted HCP approach. For example, with ‘compliant, rigorous or even obsessive patients’ (Participant, 11), the HCP should be careful not to generate anxiety. These patients would perfectly apply the recommendations provided by the HCP. The HCP should rather provide flexibility and dedramatize.

Shy, nonexpansive or introverted patients have difficulty expressing themselves in groups, which calls for a specific group management (e.g., do not take turns interviewing so that the patient does not feel questioned). However, patients with extroverted personalities must be channelled by HCPs because they ‘can sometimes suffocate other patients in group sessions’ (Participant, 1).

#### 3.1.3 Need to adjust to the patient's cognitive abilities and to language barriers

The notion of speech‐language disorders (aphasia, dysarthria) was never addressed in our data. Cognitive abilities (i.e., the patient's level of understanding of educational contents as perceived by the HCPs) were seen as a prerequisite for improving an understanding of the disease and its management and being autonomous in healthcare. When HCPs perceived poor cognitive abilities, they usually reduced the content or adapted the intervention:

• There are people with whom we will not go into as much detail because we will realize that they will not necessarily understand everything. (Participant, 11)
• We help them, but without denigrating. (Participant, 9)
• We go to the essential. (Participant, 15)
• We take time… and respect them with their limits. (Participant, 25)

One programme even proposed individual rather than group sessions for patients who ‘may not understand everything’ (Participant, 14).

Language barriers (i.e., when patients are not fluent in French) also require accommodations (e.g., practical demonstrations or accompaniment by a relative able to translate).

### 3.2 How to individualize TPE

Leading a group session differed from leading an individual session: the way in which the different topics are discussed tends to be guided by the patient in individual sessions, whereas in a group, discussions remain focused on the topic of the session to avoid tangents and special discussions outside the group.

In individual sessions, of course, we are more focused on the patient and therefore we will go more in the direction of the patient, whereas when we are in a group, we will work more on a theme. (Participant, 7)

Therefore, the underlying fundamental and practical question is how to individualize in a group session:

It's individual in collective. (Participant, 25)

#### 3.2.1 Time

If the aims of TPE programmes are to work on intrapersonal change processes and targets, sessions can be delivered in groups, even if individualization seems less natural than in face‐to‐face interviews. Knowing the patient and entering into an individualization process in collective sessions require time.

#### 3.2.2 Organization of the programme

The organization of the programme can help customize the intervention by formalizing the initial assessment or by the systematic addition of an individual session. TPE can also be extended at the end of group sessions with informal individual discussions. Flexibility and adaptations of the programme as individuals proceed are important principles of TPE and allow for tailoring.

#### 3.2.3 Initial assessment

Initial assessment is the keystone of patient education from which individualization of education can proceed. Aspects addressed during the initial assessment go far beyond the strict framework of the disease, its treatments or health practices to integrate psychological, social, economic or daily life aspects (e.g., dealing with elevators, domestic animals, working hours). Except for particular safety skills learning, which cannot be individualized, initial assessment may be used to generate individualized objectives.

In two programmes, patients could choose the sessions in which they participated:

They don't have to join the full program. They can choose sessions that appeal to them more than others. (Participant, 8)

##### Formally and systematically adding an individual session

For one programme, an individual session was organized after the two first group sessions to individualize a feedback session.

##### Alternative or complementary individual sessions if needed or desired by the patient

Two programmes offered the possibility of individual sessions if group sessions are refused. In four programmes, patients could also be seen individually at the end of the group sessions.

##### If needed, involvement of an HCP not systematically solicited

According to patients' needs, professionals who do not intervene in the TPE programme may be solicited (e.g., social workers, psychologists, physicians or professionals specializing in the support, long‐term follow‐up and retention of disabled people in employment).

#### 3.2.4 Content of the programme

Individualized patients' objectives of disease management can be defined and modified during the programme by taking into account patients' lifestyles and local opportunities of their living space. Moreover, gathering information during pedagogical assessments throughout the programme can be a way to understand to what extent the patient has understood and retained pedagogical contents addressed during the programme to eventually adjust the continuation of sessions. Another important point is the possibility for patients to be followed up between sessions to respond to patients' emotional reactions if needed.

##### Define objectives during sessions' progression

Objectives should be individualized:

I try to define an objective with them at each workshop, it is their objective. (Participant, 10)

##### Consideration of the patient's lifestyle

Individualization could also be found in the proposals regarding the management of the disease according to the patients' lifestyle. The caregiver must ‘understand his or her way of life’ (Participant, 26). Proposals should also take into account local opportunities of living space (e.g., for the practice of a sport).

##### Content and activities of the sessions

In the asthma programme, support tools were individually tailored with individual follow‐up booklets that included treatments, measures and warning signs. Tools for parenteral nutrition could be as follows:

Quickly recreated for the patient when we look at the initial assessment, (…) The patient can say to himself: it was created for me, I am not a patient among 100 others. (Participant, 1)

The physical activities are adapted to the difficulties encountered by patients.

##### Pedagogical assessment

Pedagogical assessment is an evaluation realized by an HCP with a specific tool or simply by observation to check the participants' understanding. The objective is to know to what extent the patient has understood and retained content addressed in the programme to adjust the programme continuation. This position is even more pronounced for ‘safety’ skills teaching for parenteral nutrition in digestive cancers and for injections in multiple sclerosis. In contrast, an HCP considered pedagogical assessment not really appropriate because it was like being at school.

##### Individualized follow‐up

Two programmes offered patients the opportunity to call if necessary between sessions, particularly for reinsurance.

#### 3.2.5 Relationship with the patient

##### Relationship is deeper than in routine practice

The patient–HCP relationship is deeper in TPE than in usual consultation because the person is considered as a whole, with their environment, disease(s), desires, needs and tastes. Trust is built over time. The quality of the relationship is based on a bottom‐up communication from the HCPs and is centred on the patient's concerns and not on predefined contents.

##### Patient relationships are built over time

A trust and listening relationship particular to TPE is considered to be built as the sessions progress. Three nurses clearly expressed it.

#### 3.2.6 Communication style with patients

##### Not to have a masterful position in knowledge transmission

The central element in communication style with patients in TPE is not to have a masterful position in knowledge transmission and to enable patients to find appropriate solutions for themselves:

We are trying to say as little as possible. We propose a subject but we will wait for them to bring us their knowledge. (Participant, 5)

##### Focus on patients' interests

Communication should also be centred on the patient's interests:

• Not lecturing, we are not at school, even if sometimes we are forced to get into technicality, we try, (…) to help them to verbalize the solution themselves (Participant, 10)
• I try to tell them, finally what is it that makes you here today? to try to put them in an acting position and to know where are their expectations, why are they coming here. (Participant, 15)

Focusing the session on the patient's concerns seemed an essential aspect in communication for the individualization process*:*

• We asked everyone a little bit about the question(s) that concerned them the most and therefore at the end of the questions we will be able to target an element [about] which we want to talk (Participant, 10)
• I do not do it based on my idea but on what people bring to define what will be worked on during the session. (Participant, 15)
• You have to talk to them about the things that concern them. (Participant, 4)

#### 3.2.7 How to facilitate group sessions

##### Knowing the participants beforehand

HCPs' communication tricks are used to tailor the TPE intervention during group sessions particularly to focus the patient's attention on the right message at the right time. With the initial assessment or knowledge of the patient, group sessions are conducted differently depending on the composition of the group.

##### Facilitating sessions in pairs increases HCPs' attentional capacity

In groups, leading sessions in pairs increases HCPs' attentional capacity and therefore their propensity to adapt their communication to provide appropriate answers to patients.

• I admit that I will hear some things, my colleague will hear it differently, she will relaunch it differently and I am delighted because I did not hear it the same way she did and it allows even more openness, debate and listening. (Participant, 16)
• If I do not have the idea right away to answer, I have … my colleague. (Participant, 17)

Moreover, for the multipathology programme, intervening in pairs allowed for constituting working subgroups (in group session) by each pathology.

#### 3.2.8 Patient actor of the individualization process

Individualization could come from the patient, who could choose to address ‘questions or problems that have not been perceived’ (Participant, 17).

## 4 DISCUSSION AND CONCLUSION

### 4.1 Discussion

In our sample, most TPE providers were nurses, which is consistent with the French distribution of HCPs involved in TPE. Only two men were interviewed. Because the needs of support can differ by sex, the low number of men interviewed could have affected the results. However, the proportion of men in the sample is consistent with the sex distribution of HCPs providing TPE in France. The French Directorate for Research, Studies, Evaluation and Statistics (Direction de la recherche, des études, de l'évaluation et des statistiques) reports that 87% of nurses are women. For allied health professionals, the distribution is more or less in equilibrium concerning physiotherapists. However, only 6% of dieticians are men (https://drees.solidarites-sante.gouv.fr/).

Although individualization of TPE is easier in individual than group sessions, only 4 of 14 programmes proposed individual sessions. However, individualization was still possible with groups.

For all programmes in which group sessions were proposed, the individualization process in recruitment was never organized because patients were recruited progressively to constitute a group with enough participants. In other words, the segmentation proposed by Hawkins et al.^30^ with group‐targeted interventions (patients' screening to constitute homogeneous groups to deliver an adapted content) was not applicable to the real‐life care situation. Consequently, if no homogenization group strategy can be applied, HCPs are face to face with the patient or most of the time are required to individualize their intervention during group sessions. Therefore, HCPs were most of the time required to individually tailor TPE interventions in a heterogeneous group intervention.

From HCPs' point of view, individualization is important and necessary because each patient has different needs and priorities.

The HCPs therefore fully endorse the recommendations and what is known about the importance of individualization. Indeed, as underlined by the Model of Persuasion of Petty and Cacioppo's,^31^ a message is better considered if it is personally suited. Moreover, a message that meets the unique needs of a singular person will enhance motivation and desire to make life‐style changes.^32^

Then, we tried to follow Hawkins et al.'s^30^ propositions, which highlighted the need to focus research on how tailoring works.

The first step to enter into an individualization process was formalized in each programme by the patient's initial assessment, the cornerstone to know the patient and his or her environment and to generate individualized objectives. In our results, the initial assessment was the only formal element relating to individualization of programmes with only group sessions. The French National Authority for Health considers the initial assessment, called the educational diagnosis in French, as ‘essential for getting to know the patient, identifying his or her needs and expectations and formulating with him or her the skills to be acquired, mobilized or maintained’.^2^

If the initial assessment is formalized and is a request from the French National Authority for Health, our study provides insights into informal practices to individualize TPE. Indeed, ways to individualize TPE are left to the discretion of the HCPs and they have to be creative to concretely deploy an individualization process in their practice.

Several practices were identified: giving an individual more time if needed, involving a specific HCP not systematically solicited, eliciting individual objectives at the beginning and during the programme according to the patient's progression, quickly customizing programme tools and taking into account the results of pedagogical evaluations to understand the nonassimilated content and readjust subsequently.

Moreover, reinforcing the HCP–patient relationship considerations for time, avoiding asymmetrical posture, focusing on patients' concerns and leading sessions in pairs were underlined.

Finally, the idea that the patient is also the actor of the individualization process was highlighted because the patient could choose to take ownership of the sessions and be active or passive.

The framework for any medical communication obviously refers to the context of a one‐to one meeting (focusing on interaction) between a patient and a physician foremost because of medical confidentiality. However, the model from Haes and Bensing appeared to be suitable first because the fundamental question for TPE providers is how to tailor sessions for each patient in a group setting (i.e., individualized communication in groups). Second, patient‐centred communication needs to be adapted to the patient, the disease and the healthcare setting.^62^

The Haes and Bensing framework includes six functions with related endpoints: (1) Fostering the relationship: the quality of the relationship between the patient and HCP fostered with generic elements such as trust and respect; (2) Gathering information about symptoms, experience, expectations and psychosocial characteristics; (3) Providing information: clarifies the patient's health problems, is the basis for decision‐making, reduces uncertainty and supports coping efforts; (4) Involving the patient in decision‐making; (5) Enabling disease‐ and treatment‐related behaviour: behaviours included in lifestyle or treatment management are the essential component of this function; and (6) Responding to emotions inherent to the disease context: detection of emotional problems and adequate response may interfere with other functions.

**Figure 1:**
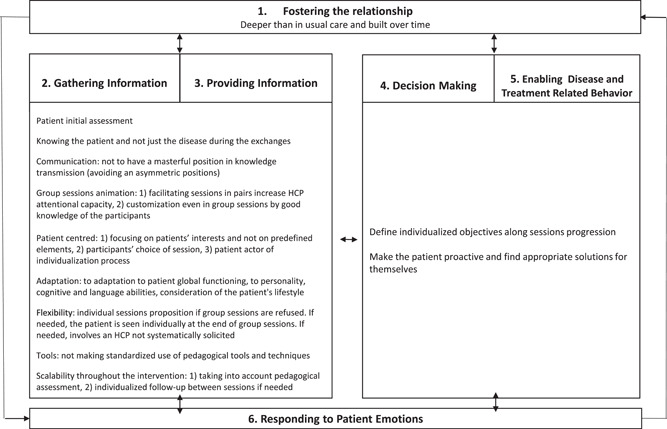
An adapted six‐function model for individualization in therapeutic patient education with related communication skills. HCP, healthcare provider

Figure 1 presents the adaptation of the Haes and Bensing framework^63^ to our study: the six functions are illustrated by our results, highlighting the tailoring process at work in individualization in TPE interventions.

Figure 1 presents an overview of how to tailor TPE for individualization in an individual configuration or in a group intervention without any strategy concerning group composition. The function Responding to emotions was organized in a transversal way with reciprocal exchange to show the fundamental aspect of this function and its relation with all others. Fostering the HCP–patient relationship is particular to TPE because the allocated time offers the possibility of an in‐depth relationship with the patient.

Gathering information is well formalized in TPE with the initial assessment, which includes information about symptoms, experience, expectation or psychosocial characteristics. However, Gathering and Providing information are both the result of an exchange of information between the patient and the HCP and were consequently merged. Apart from the formal initial assessment, the key elements in sharing information in TPE consists of (1) knowing the patient and not just the disease, (2) avoiding an asymmetric position, (3) customization even in group sessions by good knowledge of the participants, (4) focusing on patients' interests, (5) adaptation to patient global functioning and lifestyle, (6) flexibility concerning the terms of proposed sessions, (7) not making standardized use of pedagogical tools and techniques and (8) scalability throughout the intervention.

From  the thematic data analysis, we proposed a model for tailoring patient education interventions based on the Haes and Bensing medical communication model.^63^

In the initial model, the six functions were identified one after the other. However, our data seemed to suggest that in the context of TPE interventions, the ‘Responding to patient emotions’ and ‘Fostering the relationship’ were linked to each other and connected to the other four functions. We combined in one category ‘Decision making’ and ‘Enabling disease‐ and treatment‐related behaviour’ because TPE precisely aims at promoting behaviour related to lifestyle and treatment management on the basis of decisions generated by the educative intervention. Individualized objectives are defined during the progression of sessions, and patients are invited to be proactive and to find appropriate solutions for themselves.

Our study produced suggestions to elicit tailoring in TPE interventions.^30^ We propose a graphical representation of an adapted six‐function model for individualization in TPE.

### 4.2 Strengths and limitations

Qualitative data were generated and analysed according to COREQ criteria; 28 of the 32 items of the COREQ checklist were fulfilled. Hence, findings were a synthesis of HCP views and experiences based on a robust qualitative study. However, no generalizable result can be provided using maximum variation sampling.^64^

Moreover, findings could have been strengthened by further adding session observations to capture some aspects of individualization implemented without HCPs' conscious knowledge. Participants did not provide feedback on findings, which would have been interesting.

### 4.3 Conclusion

The present work has paved the way for evaluation, then generation of recommendations and finally implementation of training for individualization in educational interventions. We provide a description of ‘how’ tailoring can be implemented in practice in TPE interventions. It is a prerequisite to develop further studies to answer the following questions: What aspects of tailoring work? How does tailoring work? Immediate, intermediate and long‐term outcomes within our adapted six‐function model for individualization in TPE could have different natures (patient satisfaction, patient emotional adjustment, patient treatment adherence, effective health behaviour).^63^ Moreover, a ‘functional approach has a number of pedagogical implications’.^65^ A perspective is to design a training for communication in individualization from an evidence‐based approach.

The general CONCErTO project consists of the development of a classification of TPE components to better describe programmes. Concerning the individualization process, the dimensions identified for the communication function underlying individualization will be included to describe TPE interventions in the CONCErTO classification,^63^ which will allow for moving towards evidence‐based practice in TPE. A clear investigation of these dimensions will help determine their relative effects on the outcome, participation and sustainability of a TPE programme. The practice implications are the development of communication skills training for tailoring interventions in TPE.

## CONFLICT OF INTERESTS

The authors declare that there are no conflict of interests.

## AUTHOR CONTRIBUTIONS

**Laetitia Ricci**: methodology, investigation, formal analysis and writing—original draft. **Julie Villegente**: formal analysis. **Déborah Loyal** and **Carole Ayav**: writing—review and editing. **Joëlle Kivits**: Conceptualization, investigation and formal analysis. **Anne‐Christine Rat**: formal analysis, funding acquisition and supervision.

## Supporting information

Supporting information.

## Data Availability

The data that support the findings are available on request from the authors.
